# Prediction of congenital heart disease for newborns: comparative analysis of Holt-Winters exponential smoothing and autoregressive integrated moving average models

**DOI:** 10.1186/s12874-022-01719-1

**Published:** 2022-10-01

**Authors:** Weize Xu, Zehua Shao, Hongliang Lou, Jianchuan Qi, Jihua Zhu, Die Li, Qiang Shu

**Affiliations:** 1 Department of Cardiac Surgery, The Children’s Hospital, Zhejiang University School of Medicine, National Clinical Research Center for Child Health, No. 3333 Binsheng Road, Binjiang District, Hangzhou, 310000 Zhejiang China; 2 Zhengzhou University People’s Hospital, Henan Provincial People’s Hospital, Zhengzhou, 450003 China; 3 Jinhua Maternal and Child Health Care Hospital, Jinhua, 321000 China; 4 Department of Nursing, The Children’s Hospital, Zhejiang University School of Medicine, National Clinical Research Center for Child Health, Hangzhou, 310000 China

**Keywords:** Congenital heart disease, Newborns, Time series, Holt-Winters exponential smoothing, Autoregressive integrated moving average model

## Abstract

**Objective:**

To describe the temporal trend of the number of new congenital heart disease (CHD) cases among newborns in Jinhua from 2019 to 2020 and explored an appropriate model to fit and forecast the tendency of CHD.

**Methods:**

Data on CHD from 2019 to 2020 was collected from a health information system. We counted the number of newborns with CHD weekly and separately used the additive Holt-Winters ES method and ARIMA model to fit and predict the number of CHD for newborns in Jinhua. By comparing the mean square error, rooted mean square error and mean absolute percentage error of each approach, we evaluated the effects of different approaches for predicting the number of CHD in newborns.

**Results:**

A total of 1135 newborns, including 601 baby girls and 534 baby boys, were admitted for CHD from HIS in Jinhua during the 2-year study period. The prevalence of CHD among newborns in Jinhua in 2019 was 0.96%. Atrial septal defect was diagnosed the most frequently among all newborns with CHD. The number of CHD cases among newborns remained stable in 2019 and 2020. There were fewer cases in spring and summer, while cases peaked in November and December. The ARIMA(2,1,1) model relatively offered advantages over the additive Holt-winters ES method in predicting the number of newborns with CHD, while the accuracy of ARIMA(2,1,1) was not very ideal.

**Conclusions:**

The diagnosis of CHD is related to many risk factors, therefore, when using temporal models to fit and predict the data, we must consider such factors’ influence and try to incorporate them into the models.

## Introduction

Congenital heart disease (Congenital heart defect, CHD), one of the most common birth defects among perinatal infants, has caused great harm to health and life [1–3]. CHD includes a great number of types, such as holes inside the heart that make the blood unable to flow normally. In some cases, CHD could be detected at birth. And other times, these problems may not be discovered until after adulthood [4]. In 2015, 48.9 million people worldwide were reported to have CHD [5]. CHD is one of the leading causes of birth defect-related deaths, it resulted in more than 300,000 deaths in 2015 [6]. The incidence of CHD is usually higher in developing countries than in developed countries [7, 8]. The prevalence of CHD in Beijing was about 7.77 per 1000 births in 2016 and a total of 1851 newborns were diagnosed with critical CHD during 2010–2017 in Beijing, the prevalence was 10.43 per 10,000 [1, 9]. Previous studies have identified that genetic and environmental factors are risk factors for CHD [10]. However, there is no effective method to prevent CHD.

Time series are arranging the numeric value of statistical indicators in chronological order and forming corresponding sequences. When study on the time series of certain infectious diseases or disease events, the long-term trends, seasonal patterns, cyclic or rhythmic patterns of them allow for modelling and prediction of future outbreaks. For decades, the temporal models have been greatly developed and can be divided into deterministic models and stochastic models. Deterministic models are usually suitable for time series with typical variation characteristics. While the data of infectious diseases do not always have some typical variation characteristics, which makes the stochastic error terms produced by deterministic models cannot meet the conditions for randomness. Therefore, researchers usually choose stochastic models rather than deterministic models to perform the time-series analysis for disease events. Based on the temporal models, time-series analysis has been widely used in epidemiology to fit the data, such as influenza, malaria and so on. Spaeder et al. built a Box-Jenkins model using laboratory-confirmed H1N1 influenza incidence data in 2009 to forecast the H1N1 incidence during 2010–2011 [11]. The result showed that the 95% confidence intervals (95% CI) of the Box-Jenkins model were accurate to ±3.6 cases per 3-day period for their institution, which suggest this model may be a useful tool in forecasting the incidence of H1N1 influenza. Alegana et al. used a Bayesian Spatio-temporal conditional-autoregressive model to fit the malaria data in Afghanistan from 2006 to 2009 [12]. They found that the incidence of malaria usually peaked in August and November, this discovery would make a great contribution to the malaria case management in a local area. To determine the possible trend and seasonal pattern in hospitalizations for pulmonary embolism (PE) in Spain, Guijarro et al. used some different kinds of methods to generate a predictive time series model, which showed a linear increase and a seasonal pattern of PE incidence for hospitalizations [13].

We explored different approaches including the exponential smoothing method (ES) and autoregressive integrated moving average model (ARIMA) to fit the weekly cases of CHD among newborns in Jinhua, Zhejiang Province during 2019–2020, and then forecast the weekly cases of CHD among newborns for 3 months (12 weeks). We hypothesize a suitable temporal model which can provide a reference for the study of the epidemic trend of CHD among newborns in Jinhua, Zhejiang Province and help the government to take rational measures for disease prevention.

## Methods

### Study area and data on CHD

This study was conducted in Jinhua city, the fourth largest advanced economy region of Zhejiang Province, China. Jinhua is located in the middle of Zhejiang Province, with a total area of 10,942 km^2^. According to the official population statistics, the permanent population of Jinhua is 7,050,683 in 2020.

We collected neonatal data from all hospitals in Jinhua from 2019 to 2020 through the health information system (HIS). Diagnosis and classification of CHD for newborns were performed by qualified physicians based on ultrasound results. Newborns with CHD were classified using a previous algorithm which classified CHD based on embryo-associated defect phenotypes [14, 15]. These defect phenotypes mainly included patent ductus arteriosus (PDA), atrial septal defect (ASD), ventricular septal defect (VSD) and patent foramen ovale (PFO). Other phenotypes were uniformly classified as other due to the small number of cases. Population data was collected from Jinhua Statistic Yearbook.

### Statistical analysis

We counted the number of newborns with CHD weekly and separately used ES method and ARIMA model to fit and predict the number of CHD for newborns in Jinhua.

ES, which was put forward by Robert G. Brown, is a common method in production forecasting, also used for medium and short-term economic development trend forecasting. The basic principle of ES method is to give different weights to the observed values of the time-series data. Compared with the earlier data, the recent data will be given greater weight, by which it can better eliminate the influence of noise and get a more reasonable and reliable model. According to the counts of smoothing process and parameters, the ES method can be divided into the basic exponential smoothing method, double exponential smoothing method and triple exponential smoothing method [16–18]. The basic exponential smoothing method is to apply exponential smoothing only once for training data. The double exponential smoothing method, which applies exponential smoothing two times, is usually suitable for the time series with a linear trend. Compared with the basic exponential smoothing method and double exponential smoothing method, the triple exponential smoothing method, which applies exponential smoothing three times, incorporates the seasonal effects into the model. If we set α as the smoothing factor (0 < α < 1), then we can find that:

$${S}_t=a\times {y}_t+\left(1-a\right){S}_{t-1}$$

Where the smoothed statistic *S*_*t*_ is a simple weighted average of the current observation *y*_*t*_ and the previous smoothed statistic *S*_*t* − 1_. Therefore, basic exponential smoothing method, double exponential smoothing method and triple exponential smoothing method can be expressed respectively as:

$${\displaystyle \begin{array}{c}{S}_t^{(1)}=a\times {y}_t+\left(1-a\right){S}_{t-1}^{(1)}\\ {}{S}_t^{(2)}=a\times {S}_t^{(1)}+\left(1-a\right){S}_{t-1}^{(2)}\\ {}{S}_t^{(3)}=a\times {S}_t^{(2)}+\left(1-a\right){S}_{t-1}^{(3)}\end{array}}$$

Double exponential smoothing model, also called linear prediction model, is given by the formulas as follow:

$${\displaystyle \begin{array}{c}{\hat{Y}}_{t+T}={a}_t+T\bullet {b}_t\\ {}{a}_t=2{S}_t^{(1)}-{S}_t^{(2)}\\ {}{b}_t=\frac{a}{1-a}\left({s}_t^{(1)}-{s}_t^{(2)}\right)\end{array}}$$

Where the original data sequence of observations is represented by *y*_*t*_, beginning at time t = 0. We use *a*_*t*_ to represent the smoothed value for time t, and *b*_*t*_ is our best estimate of the trend at time t. The output of the algorithm is now written as ${\hat{Y}}_{t+T}$, an estimate of the value of x at time t + T for T > 0 based on the raw data up to time t, α is the data smoothing factor, 0 < α < 1.

Triple exponential smoothing model, with multiplicative seasonality, is given by the formulas as follow:

$${\displaystyle \begin{array}{c}{\hat{Y}}_{t+T}={a}_t+{b}_t\bullet T+{c}_t\bullet {T}^2\\ {}{a}_t=3{S}_t^{(1)}-2{S}_t^{(2)}-{S}_t^{(3)}\\ {}\begin{array}{c}{b}_t=\frac{a}{2{\left(1-a\right)}^2}\left[\left(6-5a\right){S}_t^{(1)}-2\left(5-4a\right){S}_t^{(2)}+\left(4-3a\right){S}_t^{(3)}\right]\\ {}{c}_t=\frac{a}{2{\left(1-a\right)}^2}\left[{S}_t^{(1)}-2{S}_t^{(2)}+{S}_t^{(3)}\right]\end{array}\end{array}}$$

Autoregressive integrated moving average model (ARIMA), also called Box-Jenkins model, is a classical modelling approach for non-stationary time series. Generally, the non-stationary time series need to be converted into stationary time series, then we can build ARIMA model based on the regression of hysteresis values and the previous random error terms. According to the stability of the original sequence and the parts contained in the regression, ARIMA model is usually divided into moving average process (MA), autoregressive process (AR), autoregressive moving average process (ARMA) and ARIMA process. The model is written as ARIMA(p, d, q) where p describes the AR part, d describes the integrated part, and q describes the MA part. The ARIMA model can be expressed as follows:

$$\varnothing \left(\mathrm{B}\right){\Delta }^d{Y}_t=\uptheta \left(\mathrm{B}\right){\varepsilon}_t$$

Where *Y*_*t*_ represents the response sequence, *ε*_*t*_ represents the random error at time t, ∅(B) = 1 − ∅_1_*B* − ∅_2_*B*^2^ − … − ∅_*P*_*B*^*P*^ represents the autoregressive operator, θ(B) = 1 − θ_1_*B* − θ_2_*B*^2^ − … − θ_*P*_*B*^*P*^ represents the moving average operator, and ∅(B)∆^*d*^*Y*_*t*_ represents the correlation among the different periodic points in the same periods. When P = D = Q and they all equal to 0, the model is a simple ARIMA model.

The last 3 months (12 weeks) of the dataset were divided as test sets to evaluate the accuracy of different time series models. We use Akaike’s information criterion (AIC) to evaluate the fitting effects of each approach. The prediction effects of the models are usually evaluated by the difference between the predicted value and the actual value, that is, the error. By comparing the mean square error (MSE), rooted mean square error (RMSE) and mean absolute percentage error (MAPE) of each approach, we can evaluate the effects of different approaches for predicting the number of CHD in newborns.

Time series analyses were performed using R 3.6.3 and the results with *P* ≤ 0.05 would be considered as significant.

## Results

### General characteristics

A total of 1135 newborns, including 601 baby girls and 534 baby boys, were admitted for CHD from HIS in Jinhua during the 2-year study period. The prevalence of CHD among newborns in Jinhua in 2019 was 0.96%. Overall, there were 10 newborns with CHD per week in Jinhua. The median number of newborns with CHD was higher among baby girls than which among baby boys (6.0 vs. 5.0). Up to 31 newborns in Jinhua were diagnosed with CHD in 1 week. ASD was diagnosed the most frequently among all newborns with CHD, accounting for 81.9% of all subjects. 81.6% of CHD baby boys were diagnosed with ASD, compared with 82.0% of CHD baby girls. PDA was the second most common phenotype among newborns with CHD, accounting for 64.3% of all subjects, and the constituent ratio for baby boys and baby girls were 63.7 and 62.4%, respectively (Table 1).

**Table 1:** Weekly frequency of diagnoses for congenital heart disease in newborns in Jinhua, China, 2017–2019

	Total	Boy	Girl
Number of newborns diagnosed with CHD per week	1135	534	601
Min	0	0	0
P_25_	7.0	3.0	3.0
Median	10.0	5.0	6.0
P_75_	14.0	7.0	8.0
Max	31	15	16
Defect phenotypes (n, %)^a^			
PDA	730 (64.3)	340 (63.7)	390 (62.4)
ASD	929 (81.9)	436 (81.6)	493 (82.0)
VSD	396 (34.9)	169 (31.6)	227 (37.8)
PFO	153 (13.5)	75 (14.0)	78 (13.0)
Other	139 (3.4)	63 (11.8)	76 (12.6)

*Abbreviations*: *PDA* Patent ductus arteriosus, *ASD* Atrial septal defect, *VSD* Ventricular septal defect, *PFO* Patent foramen ovale

^a^Some newborns may have one or more defect phenotypes of CHD

### Trends of CHD

**Fig. 1:**
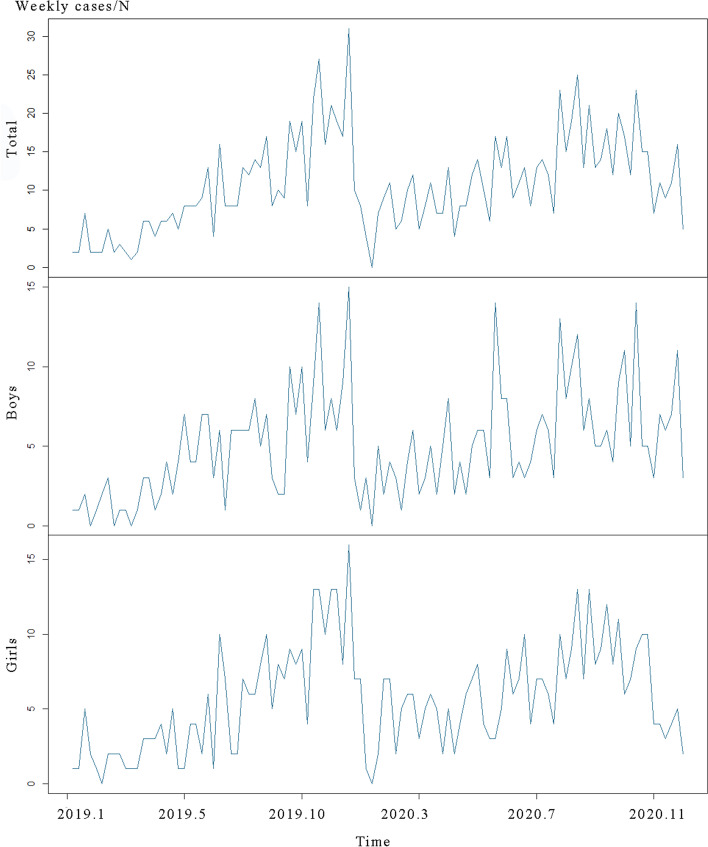
Epidemiology trend of CHD cases among newborns in Jinhua, 2019–2020

Although the duration of this study was not long enough, it could still be seen that the epidemiology trend of CHD was cyclical (Fig. 1). Overall, the number of CHD cases among newborns remained stable in 2019 and 2020. There were fewer cases in spring and summer, while cases peaked in November and December. The trend of CHD was the same in both male and female newborns as in the total subjects, with no obvious difference.

### Fitting results

**Fig. 2:**
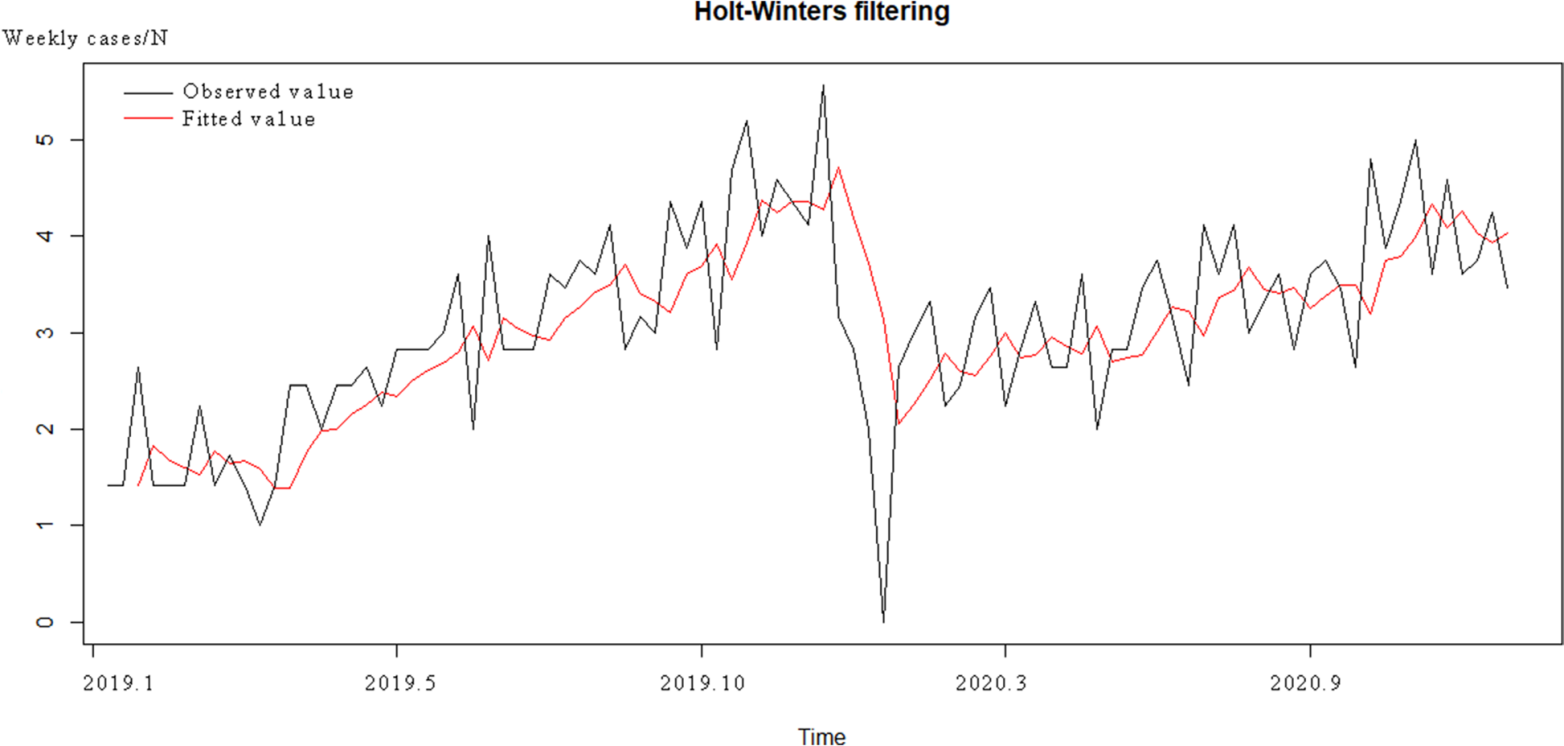
Fitting results using additive ES model

We firstly used the additive Holt-winters ES method to fit the time series data of CHD in Jinhua. The fitting result was shown in Fig. 2. The additive ES model performed well in the early stage of fitting, however, it could no longer fit the training-set data well in the later stage. The results of parameter estimation showed that this time series had no obvious seasonality and the ES model could not fit the long-term trend well. The horizontal smoothing factor, seasonal smoothing factor and trend smoothing factor were all less than 0.001 and had no significance (*P* < 0.05).

**Fig. 3:**
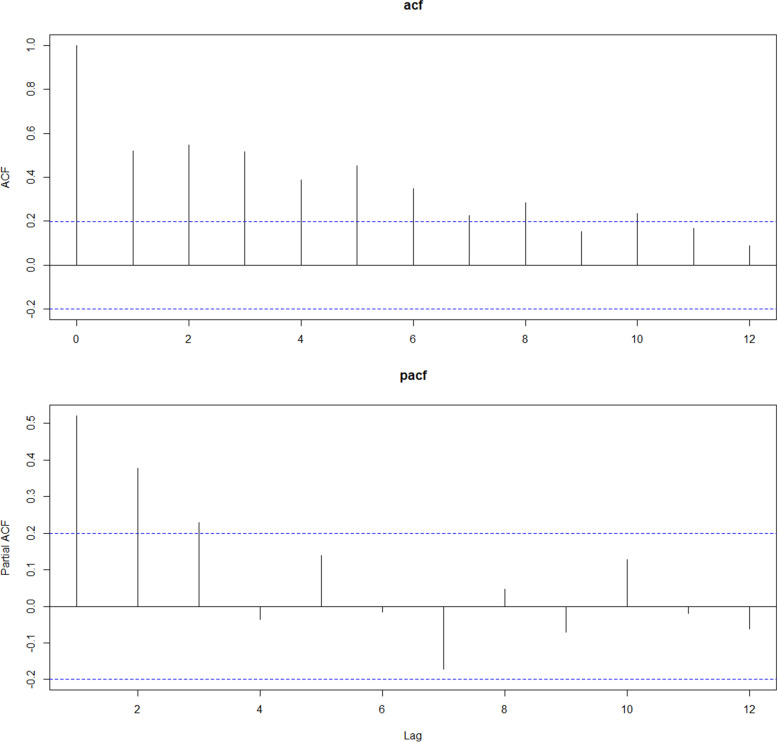
Results of auto-correlation and partial correlation after first order differencing

Then we used ARIMA model to fit the training-set data. According to the observation of the original sequence and the result of Kwiatkowski-Phillips-Schmidt-Shin test, we can find that the time series of cases with CHD in Jinhua is non-stationary (KPSS Level = 0.834, *P* = 0.01). Therefore, we did a first order differencing to make it smooth. The results of auto-correlation and partial correlation after first order differencing were presented in Fig. 3.

**Fig. 4:**
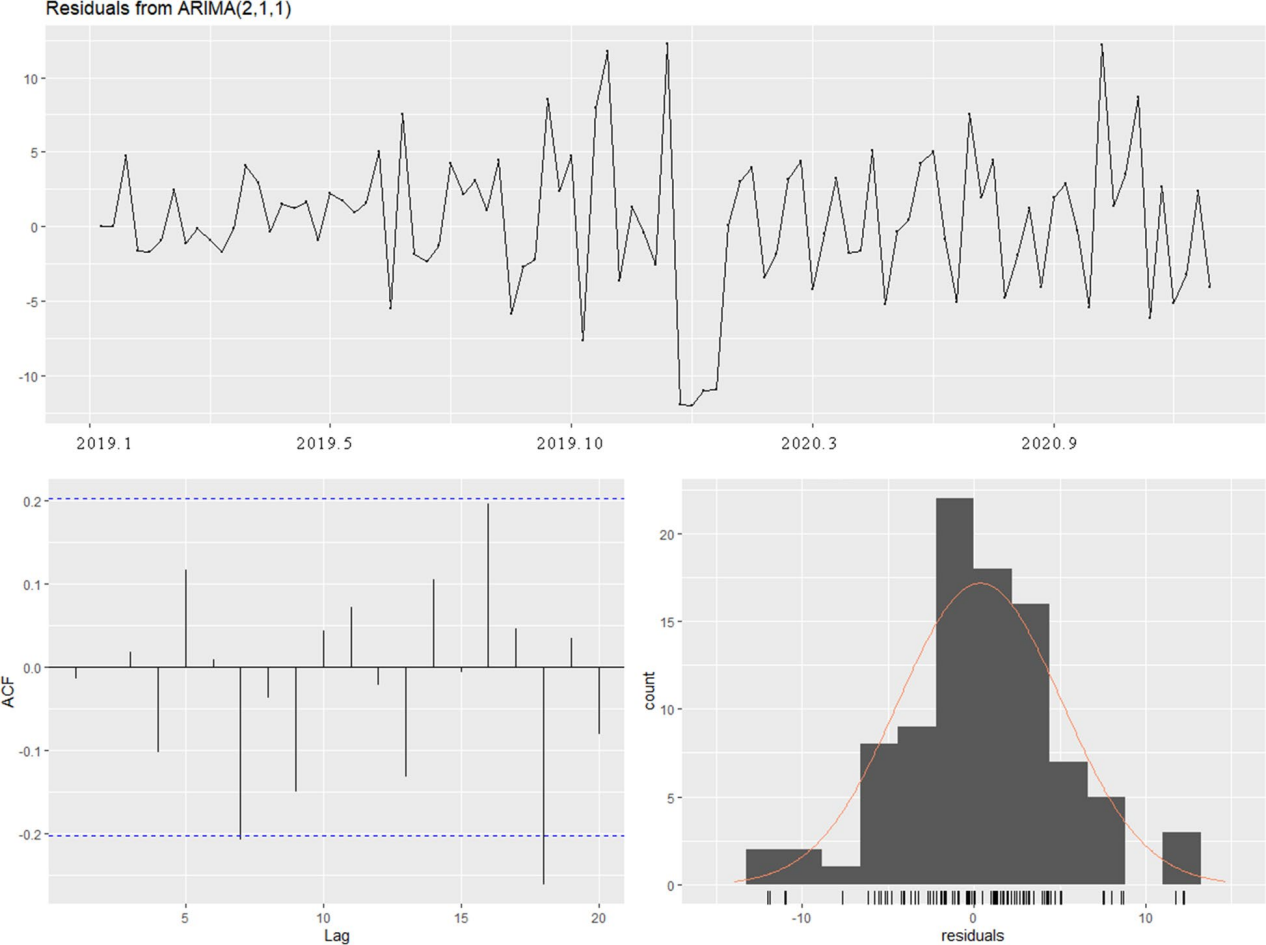
Residual of ARIMA(2,1,1) model

The results showed that after differencing the sequence is randomly fluctuating with 0-centered, which suggested that this sequence is stable. Combining the information from auto-correlation figure and partial auto-correlation figure, we finally tried to establish ARIMA(2,1,1) model. The residual of ARIMA(2,1,1) model was shown in Fig. 4. Dickey-Fuller test was used to examine the stationary.

We used least squares to build the ARIMA(2,1,1) model for the differencing sequence, the results showed that the parameters of the first order moving average model was − 0.588, and the parameters of the first order auto regression model and the second auto regression model were − 0.133 and 0.156. respectively. The AIC is 557.48, and this ARIMA(2,1,1) model can be given as follow:

$$\Delta \log (x)=\frac{\left(1+0.588B\right)}{\left(1+0.133B-0.016{B}^2\right)}{\varepsilon}_t$$

### The comparison of different models

We respectively used the additive ES model and ARIMA(2,1,1) model to forecast the weekly number of CHD cases among newborns for 12 weeks in Jinhua. Each approach’s MSE, MAPE and RMSE were calculated to compare the predictive effect (Table 2).

**Table 2:** The comparison of ES method and ARIMA model for the weekly new cases of CHD among newborns in Jinhua

Time	Original number of CHD cases	ARIMA(2,1,1)	Additive Holt-winters ES method
Last 12 week	20	15	6
Last 11 week	17	11	3
Last 10 week	12	6	3
Last 9 week	23	6	2
Last 8 week	15	4	0
Last 7 week	15	3	3
Last 6 week	7	2	3
Last 5 week	11	3	3
Last 4 week	9	3	2
Last 3 week	11	3	3
Last 2 week	16	3	3
Last 1 week	5	2	4
MSE	–	84.83	137.17
MAPE	–	226.07	245.47
RMSE	–	9.21	11.71

The results indicated that MSE, MAPE and RMSE of ARIMA(2,1,1) model were smaller than the additive Holt-winters ES method (MSE is 84.83, MAPE is 226.07 and RMSE is 9.21, respectively). We finally determine the most suitable predictive model for the study of new cases with CHD among newborns in Jinhua was ARIMA(2,1,1) model.

## Discussion

In this study, we described the temporal trend of newborns with CHD in Jinhua, Zhejiang Province from 2019 to 2020 and separately used the additive Holt-winters ES method and ARIMA model to fit and forecast the weekly number of cases with CHD among newborns in Jinhua. Totally 1135 newborns with CHD were included in this study and there was an average of 10 newborns with CHD per week in Jinhua. ASD was the most common type of CHD, accounting for 81.9% of all subjects. The weekly number of new CHD cases among newborns had a distinct peak and a slump every year and the seasonality was not obvious. The ARIMA(2,1,1) model relatively offered advantages over the additive Holt-winters ES method in predicting the number of newborns with CHD, while the accuracy of ARIMA(2,1,1) was not very ideal.

CHD is one of the most common congenital anomalies and imposes a severe emotional and economic burden on children and their families. Previous studies have shown that a range of malformations, including coronary artery disease, also described in genomic rearrangement syndromes, are difficult to diagnose in newborns [19]. The etiology of CHD remains uncertain. Maternal exposure during pregnancy is strongly associated with CHD in infants [20–22]. Some studies suggest that infection during pregnancy (e.g., German measles), exposure to toxic substances, and folic acid deficiency may be risk factors for CHD [10, 23, 24]. Since CHD usually occurs during embryogenesis, it is difficult to detect by examination during this period. Some CHD can be diagnosed prenatally by fetal echocardiography, while some CHD is usually diagnosed shortly after birth or sometimes even many years later [25].

Before this study, few studies have described and analyzed the prevalence trend of CHD in newborns. We found that the annual number of CHD patients in newborns was low at the beginning of the year, then gradually increased and peaked at the end of the year. However, no significant seasonal trends were observed. This phenomenon may be related to certain social factors in China, such as the lowest number of newborns with CHD during the Lunar New Year holiday, that is, the number of CHD among newborns is limited by the hospital’s diagnostic capacity. Our study suggests that increasing hospital capacity during the holidays may enable more newborns with CHD to be diagnosed and treated promptly on time. More data are needed to confirm and further uncover other patterns of CHD incidence among newborns.

ARIMA model is the most common method for non-stationary time series analysis, it can integrate the trend factors, long-term factors and random errors from the original sequences and extract the deterministic information by transforming the non-stationary time series into stationary time series. ARIMA model is widely used in various fields and it works well on prediction. Omar et al. extract words from article titles and propose a novel hybrid neural network model based on ARIMA model to forecast sales [26]. In Sweden, researchers use it to estimate the association between cannabis and alcohol use among teenagers [27]. Cortes et al. also try to estimate the temporal patterns of dengue incidence in two Brazilian cities through ARIMA model [28]. Besides, ARIMA model is also one of the popular machine learning techniques, it can even be used to predict the receiving waters of sewage treatment plants [29]. We tried to use ARIMA(2,1,1) models to fit the original data of new cases with CHD in Jinhua, Zhejiang Province. The error between the predicted value and the original value was relatively small which meant using this model for prediction was feasible to some extent.

ES method is also a very common method, it is intuitive, highly adaptable and easy to operate. We can forecast the future data by giving different weights to the observed values. ES method can fit the long-term trends, cyclic fluctuation and stochastic fluctuation of time series sequences. Since some observations were zero, we did not use the multiplicative ES method, but only used the additive Holt-winters ES method to fit the data. The results showed that in the predicting process for the new CHD cases in Jinhua, the prediction effect of the additive Holt-winters ES method was not as good as the effect of ARIMA model. Generally, the predicted values of the additive Holt-winters ES method were lower than the original values. It illustrated that additive Holt-winters ES method cannot fit the time series sequences perfectly if the original sequences had a sudden fluctuation, and as Fig. 1 shown, the number of CHD cases had an obvious decline at the end of the year, which might influence the effect of this model.

Our study had some limitations. Firstly, our data was relatively single, which made it impossible to fully discuss the risk factors of CHD. Secondly, our study only included data from 2019 to 2020, and more observations are needed to refine our model. Finally, our modes were relatively simple, neural network method could be used to predict the number of cases in the future. Our study also had some advantages. Firstly, we described the temporal trend in CHD among newborns, which has rarely been addressed before. Secondly, our research data were from all hospitals in Jinhua, which was reliable and covers the region comprehensively. The onset of CHD and diagnosis of CHD are related to many risk factors, therefore, when using temporal models to fit and predict the data, we must consider such factors’ influence and try to incorporate them into the models.

## Conclusions

In general, although ARIMA(2,1,1) offered advantages over the additive Holt-winters ES method in the prediction of the weekly new cases with CHD among newborns in Jinhua, Zhejiang Province, the accuracy of time series models in predicting new cases with CHD was still inadequate. More detailed information on cases should be collected and an improved time series model is necessary to predict the number of new cases with CHD among newborns in the future.

## Data Availability

The datasets used and analyzed during the current study are available from the corresponding author on reasonable request.

## Abbreviations

CHD: Congenital heart disease
95% CI: 95% confidence interval
ES: Exponential smoothing
ARIMA: Autoregressive integrated moving average
HIS: Health information system
PDA: Patent ductus arteriosus
ASD: Atrial septal defect
VSD: Ventricular septal defect
PFO: Patent foramen ovale
MA: Moving average
AR: Autoregressive
ARMA: Autoregressive moving average
AIC: Akaike’s information criterion
MSE: Mean square error
RMSE: Rooted mean square error
MAPE: Mean absolute percentage error
